# Comprehensive Analysis of Coupled Proline Cis–Trans States in Bradykinin Using ωBP-REMD Simulations

**DOI:** 10.1021/acs.jctc.3c01356

**Published:** 2024-03-11

**Authors:** Maximilian Kienlein, Martin Zacharias, Maria M. Reif

**Affiliations:** Center for Functional Protein Assemblies (CPA), Physics Department, Chair of Theoretical Biophysics (T38), Technical University of Munich, Ernst-Otto-Fischer-Str. 8, 85748 Garching, Germany

## Abstract

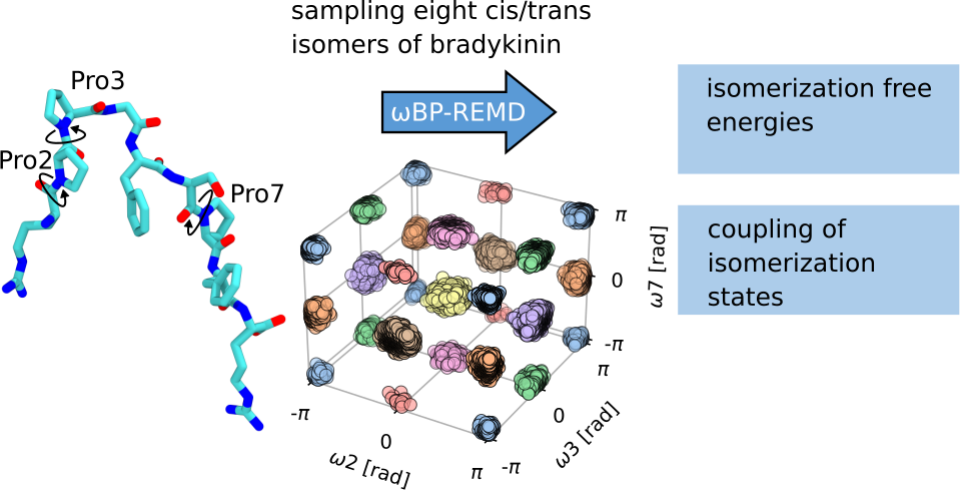

It is well-known that proline (Pro) cis–trans isomerization plays a decisive role in the folding and stabilization of proteins. The conformational coupling between isomerization states of different Pro residues in proteins during conformational adaptation processes is not well understood. In the present work, we investigate the coupled cis–trans isomerization of three Pro residues using bradykinin (BK), a partially unstructured nonapeptide hormone, as a model system. We use a recently developed enhanced-sampling molecular dynamics method (ω-bias potential replica exchange molecular dynamics; ωBP-REMD) that allows us to exhaustively sample all combinations of Pro isomer states and obtain converged probability densities of all eight state combinations within 885 ns ωBP-REMD simulations. In agreement with experiment, the all-trans state is seen to be the preferred isomer of zwitterionic aqueous BK. In about a third of its structures, this state presents the characteristic C-terminal β-turn conformation; however, other isomer combinations also contribute significantly to the structural ensemble. Unbiased probabilities can be projected onto the peptide bond dihedral angles of the three Pro residues. This unveils the interdependence of the individual Pro isomerization states, i.e., a possible coupling of the different Pro isomers. The cis/trans equilibrium of a Pro residue can change by up to 2.5 kcal·mol^–1^, depending on the isomerization state of other Pro residues. For example, for Pro7, the simulations indicate that its cis state becomes favored compared to its trans state when Pro2 is switched from the trans state to the cis state. Our findings demonstrate the efficiency of the ωBP-REMD methodology and suggest that the coupling of Pro isomerization states may play an even more decisive role in larger folded proteins subject to more conformational restraints.

## 1 Introduction

The amino acid proline (Pro) is unique among the 20 natural amino acids. Its cyclic side chain connects the C_α_ and amide nitrogen atoms. Hence, the cis and trans states of a peptide bond preceding the Pro residue, a so-called prolyl peptide bond, are sterically more similar than those in the other amino acids. The cis isomer of a prolyl peptide bond is sometimes only about 0.5 kcal·mol^–1^ less stable than the trans isomer.^1^ Thus, in fact, around 7% of all prolyl peptide bonds are found in the cis state.^2^ In contrast, nonprolyl peptide bonds have a virtually exclusive preference for the trans state. The possibility for prolyl peptide bonds to access both isomerization states combined with the very slow cis/trans isomerization rates (on the order of seconds to minutes) is the reason why certain Pro residues have exceptional biological functions, e.g., as molecular timers^3^ or a large impact on protein folding.^4^

It has been shown before that Pro isomerization and conformational changes such as protein folding may be coupled.^5^ For example, in a protein folding process, the (reversible) work that is required to isomerize a given Pro residue to its native isomerization state can be supplied by the free energy that is gained when the protein reaches a stable fold. Such a coupling was investigated by unfolding and refolding experiments of the N-terminal domain of the gene-3 protein of the phage fd.^5,6^ In previous work, we performed extensive molecular dynamics (MD) simulations to calculate Pro isomerization free energies in the wild-type and various mutant systems of this protein, as well as in corresponding unfolded-state models.^7^ In the present work, we perform Pro isomerization free-energy calculations in a peptide where multiple Pro residues occur, bradykinin (BK).

In 1981, Levitt already investigated a folded protein where multiple Pro residues occur, bovine pancreatic trypsin inhibitor, and calculated the energy differences of Pro isomerization states.^8^ It was observed that the cis/trans energy differences of the Pro residues may be very different, and their magnitude correlates with the experimentally observed impact of the “wrong” (non-native) isomerization state on the proper folding of the protein. For example, Pro13 was seen to have a very low energy difference between the cis and trans states and is supposed to be able to isomerize freely even in the folded protein, whereas Pro8 was seen to have a very high energy difference between the cis and trans states and can block the folding to the native state when it is in the “wrong” cis form.

In the absence of structural constraints, however, as is, for example, the case in short peptides or disordered proteins, it is commonly believed that a coupling between Pro isomerization states and biomolecular conformation does not occur.^9,10^ In the present work, we amend this view. For the partially unstructured nonapeptide BK, we illustrate, using the enhanced-sampling free-energy calculation method ω-bias potential replica exchange molecular dynamics (ωBP-REMD),^7^ that distinct cis/trans isomerization states of the three Pro residues in BK may sample distinct conformational clusters. We also show that the distinct cis/trans isomerization states of the three Pro residues may occur in a nonindependent fashion, i.e., the cis/trans equilibrium of a given Pro residue may depend on the isomerization states of other Pro residues. We refer to this phenomenon as the coupling of proline cis–trans isomerization states. It was observed before using ion mobility-mass spectrometry that BK conformation depends on the isomerization states of the Pro residues.^11^ Furthermore, Yang et al. performed MD simulations to sample the eight possible isomerization states of BK and noted a possible mutual dependence of the isomerization states of the individual Pro residues.^12^ However, our present work is, to our knowledge, the first study to provide detailed and complete insight into the coupling of all of the isomerization states of the Pro residues in BK by systematically quantifying the dependence of the cis/trans equilibrium of a given Pro residue on the isomerization states of the remaining Pro residues. We refer to these isomerization free energies as conditional isomerization free energies.

BK, besides being an ideal system to study the connection between Pro isomerization states and molecular conformation and/or the isomerization equilibria of the other Pro residues, is a very interesting system in structural biology because it features both ordered and disordered regions, which prevents a complete determination of its structure. Numerous experimental efforts have been made to determine BK structure in solution. Circular dichroism,^13−15^ Raman spectroscopy,^16^ nuclear magnetic resonance (NMR),^15,17−19^ and molecular modeling studies^20−22^ reveal that the BK Ser6-Arg9 residues are able to adopt a β-turn motif. The preference for the β-turn motif is enhanced by aprotic solvents, apolar media, or micelles.^23^ Thus, the extent of conformational structure in BK depends crucially on the environment. It is mainly due to the N-terminal Arg1-Phe5 region that BK is described as a partially unstructured peptide. This region appears to be a random coil-like segment with no dominant structural features. Ion mobility and mass spectrometry measurements in the gas phase provide evidence for the coexistence of different conformations in the Arg1-Phe5 region, depending on protonation states and solvent.^11,24,25^ The impact of the protonation state on the structure of BK was also observed in computational studies.^26,27^

Pro residues are key in establishing the diversity of conformations of free BK.^11^ Specifically, distinct combinations of cis or trans forms of the three residues (Pro2, Pro3, and Pro7) are responsible for some of the populations observed experimentally. Interestingly, conformers of [BK + 3H]^3+^ (meaning the peptide with a neutral C-terminus while the two arginine residues and the N-terminus are positively charged) in solution were seen to have a surprisingly high preponderance to incorporate cis Pro configurations.^11^

Concerning biological function, BK appears to be an extraordinarily versatile peptide. It possesses an astonishing ability to play diverse roles in regulating numerous physiological processes. It can bind to the endothelial G-protein-coupled receptors (GPCRs) B_1_ and B_2_ and thus initiate signal transduction to exert potent pharmacological and physiological effects ranging from blood pressure regulation, vasodilation, or pain response to inflammation.^28−30^ BK has also been shown to exert potent antithrombogenic, antiproliferative, and antifibrogenic effects,^31^ which may be exploited toward clinical benefit.

According to NMR spectroscopic studies, binding of BK to the receptor B_2_ occurs in a relatively elongated conformation, with all three Pro residues in the trans conformation and a β-turn at the C-terminus.^32,33^ Hence, interestingly, only a single BK conformer appears to be found in complex with the receptor. It is still largely unclear how other, “inactive” BK conformers may convert into conformers capable of receptor binding and whether there are other, still undiscovered receptors available for binding these conformers. Clearly, complete characterization of BK conformations in different environments is important to understand its biological role and may aid in the design of more effective receptor agonist and antagonist analogues.

Most computational models for BK presume an all-trans state. Note in this context that in the present work, we distinguish the isomerization state of a given Pro residue from the isomerization state of the peptide BK. The former can exist in two forms (cis or trans), whereas the latter can exist in eight possible forms (each Pro residue being present in the cis or trans state). Accurate computational models have to incorporate the possibility of Pro cis/trans isomerization, and the presumption of a Pro residue in the trans state might not be justified, not only for BK but also for smaller peptide systems in general. Frequently found Pro residues in neuropeptides might also indicate a biological relevance of Pro cis/trans isomerization.^34^ However, any investigation of Pro/cis trans isomerization with explicit-solvent atomistic MD simulation is hampered by the extremely long time scale on which the isomerization occurs. Hence, enhanced-sampling methods have to be used to make the process accessible on a nano- or millisecond time scale. For example, Yang et al.^12^ used a combination of metadynamics and integrated tempering sampling to sample the eight different isomerization states of [BK + 3H]^3+^ in explicit solvent in 1 μs trajectories. The all-trans state was found to be the preferred state. This contrasts with the mass spectrometry-based study of Pierson et al.,^11^ where aqueous [BK + 3H]^3+^ was found to prefer a state with Pro2, Pro3 in the trans, and Pro7 in the cis isomer. Note that the metadynamics study involved three biased reaction coordinates, i.e., the three Pro-preceding peptide bond dihedral angles. Sampling thus ultimately occurs on a three-dimensional flattened free-energy surface, which implies a tremendous computational effort to adequately capture the phase-space areas of interest. Multidimensional free-energy surfaces can, in principle, be calculated in a more compute-efficient manner using exchanges between the multiple bias potentials,^35^ such as in parallel-bias metadynamics.^36^ However, the computational efficiency of this method, especially the filling-up rate, is very parameter-sensitive. In addition, diffusion through the high-dimensional reaction-coordinate space may be slow, such that it takes a long time to adequately sample all states of interest. The latter problem may, however, be overcome with selective temperature increases of fictitious degrees of freedom coupled to the reaction-coordinate variables.^37^

In the present study, we use a newly devised enhanced-sampling approach, ωBP-REMD,^7^ to quantitatively investigate coupled Pro isomerization processes in aqueous, zwitterionic BK. Our approach, based on Hamiltonian replica-exchange simulations, allows simultaneous cis/trans exchanges of all three Pro residues in BK. By equally focusing the sampling in the lowest replica on all the physically important regions of phase space, which is achieved through an additional bias potential on each of the three prolyl peptide bond dihedral angles, this method can accurately capture rarely occurring cis/trans isomerization states and reveal potential dependencies between the different isomerization states. There are two crucial differences in comparison to metadynamics, which entail the excellent computational efficiency of our method: first, sampling in the lowermost replica does not occur on a flattened free-energy surface. This means that the sampling in this replica is strongly focused on the interesting phase-space areas, namely, the cis and trans states of each prolyl peptide bond dihedral angle. Second, rather than having to sample the volume of the three-dimensional conformational space spanned by the three combined reaction coordinates, as in metadynamics, our method is based on independent biasing of the three reaction coordinates. This implies a great reduction in compute effort compared to a standard metadynamics approach.

The main objective of the present work was to apply ωBP-REMD simulations to BK to enhance the isomerization events of its three Pro residues and thus elucidate the interdependence of the three cis/trans equilibria. Our simulations of zwitterionic BK in water reveal that:

1. Distinct conformational clusters are sampled by the different isomerization states of BK.
2. The all-trans state is the preferred state, and in about one-third of the simulation frames, it adopts a C-terminal β-turn motif presenting the characteristic Ser6-Arg9 hydrogen bond.
3. Isomerization of the three Pro residues can occur in a coupled fashion, i.e., the isomerization state of a given Pro residue can influence the cis/trans equilibrium of other Pro residues.
4. The influence of Pro isomerization states on the cis/trans equilibrium of other Pro residues may be large, i.e., conditional isomerization free energies may differ by up to 2.5 kcal·mol^–1^.

Notably, to our knowledge, this is the first time that a strong coupling of Pro isomerization states has been illustrated and quantified via conditional isomerization free energies. In addition, it is noteworthy that such coupling is observed in a system like BK, i.e., a short, flexible peptide chain devoid of significant conformational restraints. This suggests that the coupling of Pro isomerization states may be even more pronounced in systems possessing more conformational restraints, e.g., as represented by defined secondary structure elements in folded proteins.

## 2 Methods

All MD simulations were performed with the CUDA-accelerated PMEMD version of the AMBER20 software suite.^38^ For the peptide, the ff14SB force field^39^ was used. Ions were described with the parameters of Joung and Cheatham.^40^ Water was represented explicitly with the TIP3P water model.^41^

### 2.1 ωBP-REMD: Simulation Setup and Details

The sequence of the peptide BK is RPPGFSPFR. The crystal structure of B_2_-receptor-bound BK (PDB ID: 6f3w)^42^ served as the starting structure for MD simulations. The N- and C-termini of the peptide were charged NH_3_^+^- and COO^–^-groups, respectively, and the two arginine residues were protonated, resulting in a net charge of +2*e*. This charge state is used for all simulations in the present study. The peptide was solvated in an octahedral box with a minimum distance of 10 Å to the box boundaries. Sodium and chloride ions were added to neutralize the system and reach an ion concentration of 100 mM. After an energy minimization of 2000 steps of steepest descent, the system was gradually heated up to 300 K while keeping positional restraints on the peptide non-hydrogen atoms. The restraints were gradually released during another simulation of 1 ns in length, followed by an unrestrained equilibration simulation of 20 ns at a constant pressure of 1 bar. Temperature and pressure were maintained at the target values by weak coupling,^43^ using coupling times of 0.1 and 2.5 ps, respectively. A nonbonded cutoff of 9 Å was used to truncate Lennard-Jones interactions and real-space interactions in the PME method.^44^ All bonds were kept at their optimal lengths using the SHAKE algorithm.^45^ Newton’s equation of motion was integrated numerically with the Velocity-Verlet algorithm.^46^ The hydrogen mass repartitioning scheme was used, allowing a time step of 4 fs.^47^

**Figure 1:**
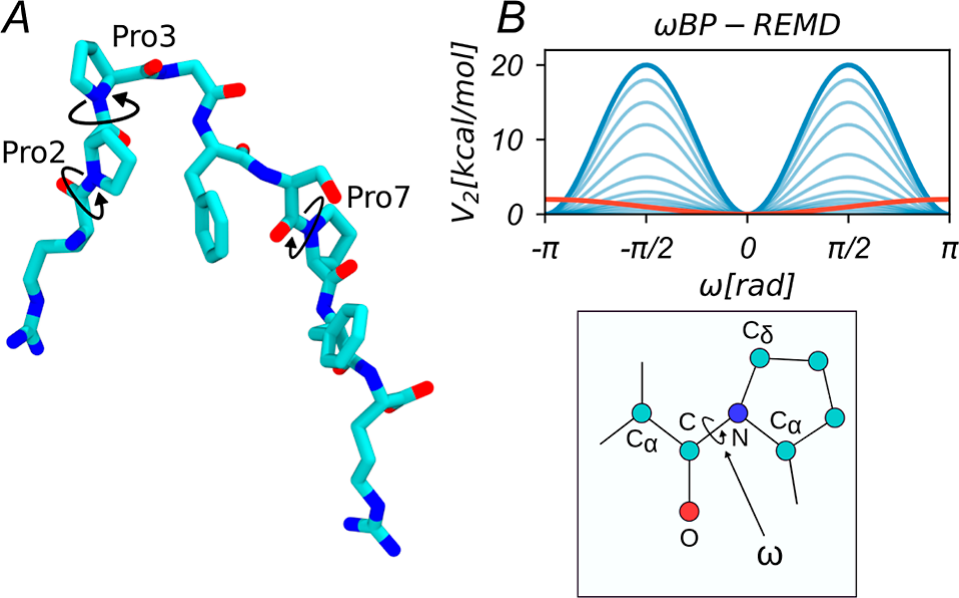
(A) Zwitterionic nonapeptide BK with Pro residues at positions 2, 3, and 7, shown in stick representation in an arbitrary conformation observed during the performed MD simulations. Torsions around the respective prolyl peptide bonds are highlighted. (B) The ω dihedral angle of Pro residue *i* is given by the atoms C_α,*i*–1_–C_*i*–1_–C_α,*i*_–N_*i*_ (bottom). ωBP-REMD simulations are done with simultaneous modification of the dihedral angle potentials of all three prolyl peptide bonds. Replicas 1 to 12 have Hamiltonians with a decreasing barrier height of Pro cis/trans isomerization (blue lines). The Hamiltonian for replica 1, while containing the physical dihedral angle energy profile with a barrier height of 20 kcal·mol^–1^ (eq 1), has an additional 1-fold cosine term (eq 2; red line) that penalizes the trans state of all Pro residues. This 1-fold cosine term is also present in the Hamiltonian of the other replicas (top).

Based on the equilibrated structure, ωBP-REMD simulations, according to our previous work,^7^ were started. The setup included 12 replicas differing in the force constant *V*_χ,2_ of the dihedral angle potentials for the dihedral angles surrounding the prolyl peptide bonds of each of the three Pro residues of BK (Pro2, Pro3, and Pro7). Equidistant spacings from *V*_χ,2_ = 0 kcal·mol^–1^ in replica 12 up to *V*_χ,2_ = 2.5 kcal·mol^–1^ in replica 1 were chosen for each of the four torsion-energy terms pertaining to the four dihedral angles surrounding each prolyl peptide bond, resulting in the effective potentials, as shown in Figure 1B. This means that unhindered Pro cis/trans transitions are possible in replica 12, whereas replica 1 represents the physical dihedral angle potentials with barrier heights of 20 kcal·mol^–1^, i.e.
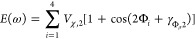
1where ω denotes the respective prolyl peptide bond angle (C_α,*i*–1_–C_*i*–1_–N_*i*_–C_α,*i*_; for residue *i* referring to Pro2, Pro3, or Pro7), the sum runs over all four Φ_*i*_ denoting the four involved dihedral angles (C_α,*i*–1_–C_*i*–1_–N_*i*_–C_α,*i*_, O_*i*–1_–C_*i*–1_–N_*i*_–C_α,*i*_, O_*i*–1_–C_*i*–1_–N_*i*_–H_*i*_, or C_α,*i*–1_–C_*i*–1_–N_*i*_–H_*i*_), and  is the phase shift.

By exchanges between replicas according to the Metropolis criterion,^48^ the respective configurations propagate into replica 1 and enhance the sampling of cis/trans isomerization in this replica. To achieve a balanced sampling of the cis/trans isomers of each Pro residue, a small potential-energy term with a cosine form of multiplicity *n* = 1, force constant *V*_ω,1_ = 1 kcal·mol^–1^, and phase shift γ_ω,1_ = 180°,
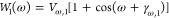
2was added in all replicas to adjust the cis/trans equilibria and destabilize the otherwise favored all-trans state of BK (Figure 1B, red line).

Each replica was simulated for 885 ns, with exchange attempts^48^ between neighboring replicas occurring every 250 steps. Acceptance rates between neighboring replicas were in the range of 28–89%.

During the ωBP-REMD simulations, coordinates were written to file every 40 ps for subsequent analysis.

### 2.2 Free-Energy Calculation

Eight possible cis/trans isomerization states involving the three Pro residues Pro2, Pro3, and Pro7, can be defined for BK. The probabilities for BK to adopt any of these states were calculated from the sampling observed in the trajectory of replica 1. The three-dimensional space describing the three Pro ω dihedral angles (ω_2_, ω_3_, and ω_7_), each ranging from −180 to 180°, was binned into cubes with 50 bins along each axis to obtain the biased probability density . The probability is biased because the physical Hamiltonian in replica 1 was augmented by the additional potential energy contribution given in eq 2. *P̃* was then reweighted via^49^
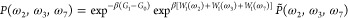
3where , *k*_B_ is Boltzmann’s constant, *T* is the absolute temperature, and *G*_1_ is, to within an additive constant, the free energy associated with the biased sampling in replica 1
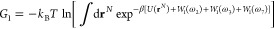
4and *G*_0_ relates to the free energy associated with the sampling in the unbiased system
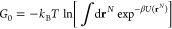
5where the integration is done over the 3*N*-dimensional vector **r**^*N*^ containing the coordinates of all atoms and *U*(**r**^*N*^) is the physical potential energy of the system. Note that kinetic energy contributions are omitted throughout because only changes in configurational variables are considered, and pressure–volume contributions are neglected due to their generally small magnitude.

**Figure 2:**
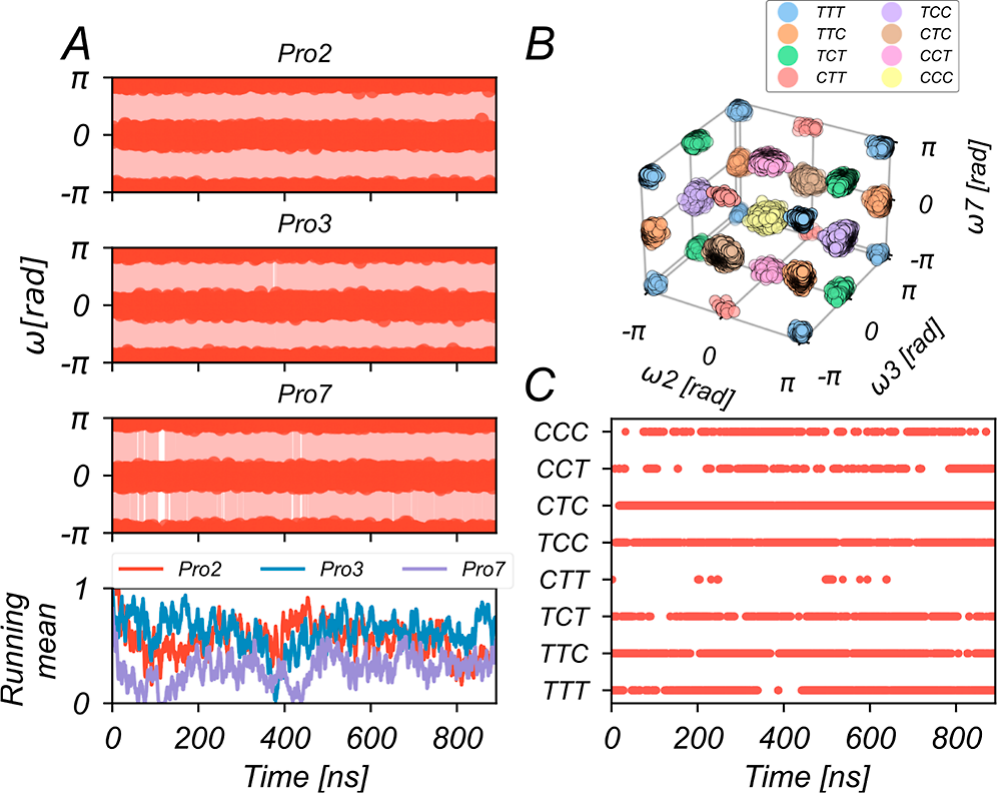
Cis/trans isomerization of the Pro residues (Pro2, Pro3, and Pro7) in BK investigated with the ωBP-REMD method. (A) Time series of the three ω dihedral angles sampled in replica 1 during a ωBP-REMD simulation of 885 ns length (top). The data is also depicted as a running mean of sampled states (eq 13), where trans states have been assigned a “1” and cis states a “0” (bottom). (B) Illustration of the isomerization states, sampled in replica 1 in the three-dimensional space given by values of the ω dihedral angle of Pro2, Pro3, and Pro7 (ω_2_, ω_3_, and ω_7_, respectively). Each dot represents a sampled simulation frame. The isomerization states of BK are labeled TTT, TTC, TCT, CTT, TCC, CTC, CCT, and CCC, where the letters “T” and “C” refer to the trans and cis isomerization states, respectively, of single Pro residues, and the first, second, and third positions refer to Pro2, Pro3, and Pro7, respectively. (C) Time series of the isomerization states of BK sampled in replica 1.

Proper normalization of *P* in eq 3 was ensured by the condition
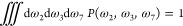
6The probabilities for each isomerization state were calculated by integrating the probability density over the corresponding ω values. The integration was done numerically using Simpson’s rule, and the dihedral angle range of the trans state was defined as 180 ± 90° and that of the cis state as 0 ± 90°. This coarse criterion is adequate to define the isomerization states in replica 1 because cis/trans states are well separated in replica 1 at ±90° (Figure 2B, 4, and 5). Free energies were computed via Boltzmann inversion
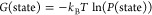
7where “state” refers to the triple ω_2_, ω_3_, and ω_7_ residing in one of the possible eight isomerization states of BK. Two- or one-dimensional probability densities and corresponding free-energy surfaces were obtained in a similar fashion by projection
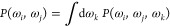
8and
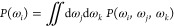
9where *i*, *j*, and *k* refer to either of the three Pro residues, Pro2, Pro3, and Pro7.

### 2.3 Conditional Isomerization Free Energies

Based on the one-dimensional probability densities (eq 9) for a Pro residue *i*, the potential of mean force (PMF) along the concerned ω dihedral angle reaction coordinate can, in principle, be obtained by Boltzmann inversion
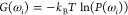
10The isomerization free energy for a cis-to-trans transition of Pro residue *i*
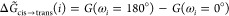
11refers to an ensemble average over the isomerization states of the remaining Pro residues *j* and *k*.

Here, we define the conditional isomerization free energy
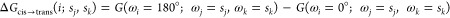
12as the isomerization free energy of Pro residue *i* with the remaining Pro residues *j* and *k* residing in defined isomerization states *s*_*j*_ and *s*_*k*_, respectively. This allows a detailed and systematic analysis of the influence of the isomerization state of different Pro residues on the isomerization equilibrium of another Pro residue of interest. Note that in practice, ranges of ω dihedral angle values of ±90° around the values of 0 and 180° were used to define a certain state.

### 2.4 Further Analyses

The sampling of isomerization events was analyzed by the running mean of isomerization states over 100 consecutive simulation frames
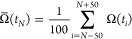
13where variable *t*_*i*_ denotes a particular simulation time point, Ω(*t*_*i*_) = 1, if ω(*t*_*i*_) of the considered Pro residues pertains to the trans state and Ω(*t*_*i*_) = 0, if ω(*t*_*i*_) pertains to the cis state. Since frames were written to file every 40 ps, a value of  close to 0.5 indicates balanced sampling of cis and trans states in a 4 ns time interval centered around *t*_*N*_.

A clustering of the sampled configurations in replica 1 was done by hierarchical density-based clustering using the HDBSCAN module of scikit-learn.^50^ This clustering was done in a combined fashion, i.e., on the entire trajectory of replica 1 involving all possible cis/trans isomerization states. Input features were selected by using hydrogen-bond contacts that are best suited for discrimination between the conformations sampled by different isomerization states. This was done by first identifying all formed hydrogen-bond contacts during the simulation of replica 1 using the hbond module of pytraj.^51^ Hydrogen bonds were defined by a donor-hydrogen-acceptor angle of at least 135° and a donor–acceptor distance of less than 3 Å. The distances *d* between donor and acceptor atom pairs were scaled according to *d*_scaled_ = (*d* – 1.8 Å)^2^ and then used as input features to train a random forest classifier with the different classes corresponding to the eight possible isomerization states of BK. The scaling proved to be helpful for the subsequent clustering as lower distances get a higher weight and the density of higher-distance noise gets reduced. As before, the scikit-learn software^50^ was used. In order to find distinct conformations that may possibly be exclusively populated by a particular BK isomerization state, the distances *d*_scaled_ were ranked by feature importance to find those that are best suited for discrimination among the eight isomerization states of BK. These distances were subsequently used as inputs for HDBSCAN clustering. The minimum cluster size was set to 500. All found clusters were validated by root-mean-square-deviation (rmsd) analysis (Figures S1–S3A). Note that we found the present approach of clustering based on important hydrogen-bond contacts to be more suited for clustering a partially unstructured peptide than a standard backbone rmsd-based approach. This is due to the large variety of backbone conformations accessible to aqueous BK, in particular concerning the random coil-like N-terminal region.

For additional analyses, the trajectory of replica 1 was filtered with respect to the eight different isomerization states of BK using dihedral angle ranges of 180 ± 45° for the trans state and 0 ± 45° for the cis state. Henceforth, we use a three-letter representation of BK isomerization states, where the letters “T” and “C” refer to the trans and cis isomers of a given Pro residue. The first letter represents the isomerization state of Pro2, the second letter represents that of Pro3, and the third letter represents that of Pro7. For instance, the all-trans isomer of BK is then denoted as TTT. The resulting trajectories for states TTT, TTC, TCT, CTT, TCC, CTC, CCT, and CCC contained 3236, 3687, 1845, 92, 3565, 6857, 1353, and 1642 frames, respectively, and allowed independent investigation of the different isomerization states.

Molecular-mechanics generalized-born surface-area (MMGBSA) free-energy calculations were performed on the different states using the MMPBSA.py software from the AmberTools software package.^52^ The implicit water model igb = 5 was employed in combination with the mbondi radii set.^53,54^ A salt concentration of 0.1 M was used, the solvent relative dielectric permittivity was set to 80, the solute interior dielectric permittivity was set to 1, and nonpolar solvation-free energy contributions were calculated^55^ based on a surface tension coefficient of 0.0072 kcal·mol^–1^·Å^–2^. Entropy calculation was omitted due to the high computational demand and the introduction of large statistical uncertainties. Energies were decomposed on a per-residue basis for further insight.^52^

The different states were also analyzed with respect to violations of the upper bounds of interproton distance ranges corresponding to measured nuclear Overhauser effect (NOE) intensities for zwitterionic BK in dimethyl sulfoxide containing 1% water at 300 K.^19^ The violation Δ_*ij*_ for a proton pair *i*, *j* was calculated as the inverse 6th-power averaged proton–proton distance, minus the upper bound *d*_*ij*_^NOE,exp^ of the experimental NOE
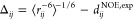
14where *r*_*ij*_ are instantaneous distances between protons *i* and *j*, and the angular brackets denote ensemble averaging over the trajectories pertaining to the different isomerization states. The upper bounds were set to *d*_*ij*_^NOE,exp^ = 3, 3.5, 4, or 4.5 Å for strong, medium, weak, or very weak NOEs, respectively.

Visualization of structures and trajectories was performed using VMD.^56^

Throughout, errors on free energies were calculated as the standard error of the mean of values from samples obtained by dividing the data in 5 equally sized subsets.

## 3 Results and Discussion

### 3.1 Enhanced Sampling of Cis/Trans Isomerization by ωBP-REMD

The ωBP-REMD scheme was capable of inducing frequent cis/trans transitions in all of the Pro residues of BK. Enhanced isomerization rates in the upper replicas propagated down the replica ladder. Thus, excellent cis/trans transition rates of 11.3, 10.6, and 9.6 ns^–1^ for Pro2, Pro3, and Pro7, respectively, were observed in replica 1 (Figure 2A, top). Enhanced sampling of isomerization events is important because it allows a statistically meaningful analysis of the cis/trans equilibrium via Boltzmann inversion of corresponding probabilities (eqs 3 and 7; Section 2.2).

The barrier height *V*_ω,1_ = 1 kcal·mol^–1^ of the added cosine term with multiplicity *n* = 1 (eq 2; Section 2.1) is suitable to achieve balanced cis–trans sampling in all three Pro residues. This is evidenced by the measure of the running mean of isomerization states (eq 13; Section 2.4), where trans states are mapped to “1” and cis states are mapped to “0”. The running mean of isomerization states indicates a small equilibration time before stable fluctuations around 0.5 without slow drifts toward any isomerization state during more than 800 ns of simulation time occur (Figure 2A, bottom).

Even if the sampling of cis/trans isomerization of any Pro residue in BK is drastically enhanced compared to an unbiased simulation setup, the fact that three Pro residues occur warrants further diagnostics to analyze the extent of sampling. For example, it could be that cis/trans isomerization events of the Pro residues are heavily correlated, meaning that, for example, only states TTT and CCC of all the eight possible isomerization states of BK would be accessed if all Pro residues always switched simultaneously between the isomerization states from trans to cis and back. Here, however, isomerizations of the different Pro residues occur with only marginal linear correlations, as shown by the very low Pearson correlation coefficients, *R*(Pro2,Pro3) = −0.14, *R*(Pro2,Pro7) = 0.29, and *R*(Pro3,Pro7) = −0.15.

Thus, all of the eight possible isomerization states of BK have been visited in multiple back and forth transitions, as shown in Figure 2B,C. The percentages of simulation time in replica 1 spent in the CCC, CCT, CTC, TCC, CTT, TCT, TTC, and TTT states are 7.4, 6.1, 30.8, 16.0, 0.4, 8.2, 16.6, and 14.5%, respectively. Although state CTT is accessed rather infrequently, the fact that its occurrences are distributed throughout the total simulation length of 885 ns supports simulation convergence and allows meaningful extraction of free energies.

In summary, ωBP-REMD allows exhaustive sampling of the important phase-space regions in replica 1 for the investigation of cis/trans equilibria in BK, even in the multidimensional isomerization process with three degrees of freedom, the peptide bond dihedral angles of Pro2, Pro3, and Pro7. Configurations that would be very unfavorable in unbiased simulations were sampled extensively.

### 3.2 Free Energy of BK Isomerization States

**Figure 3:**
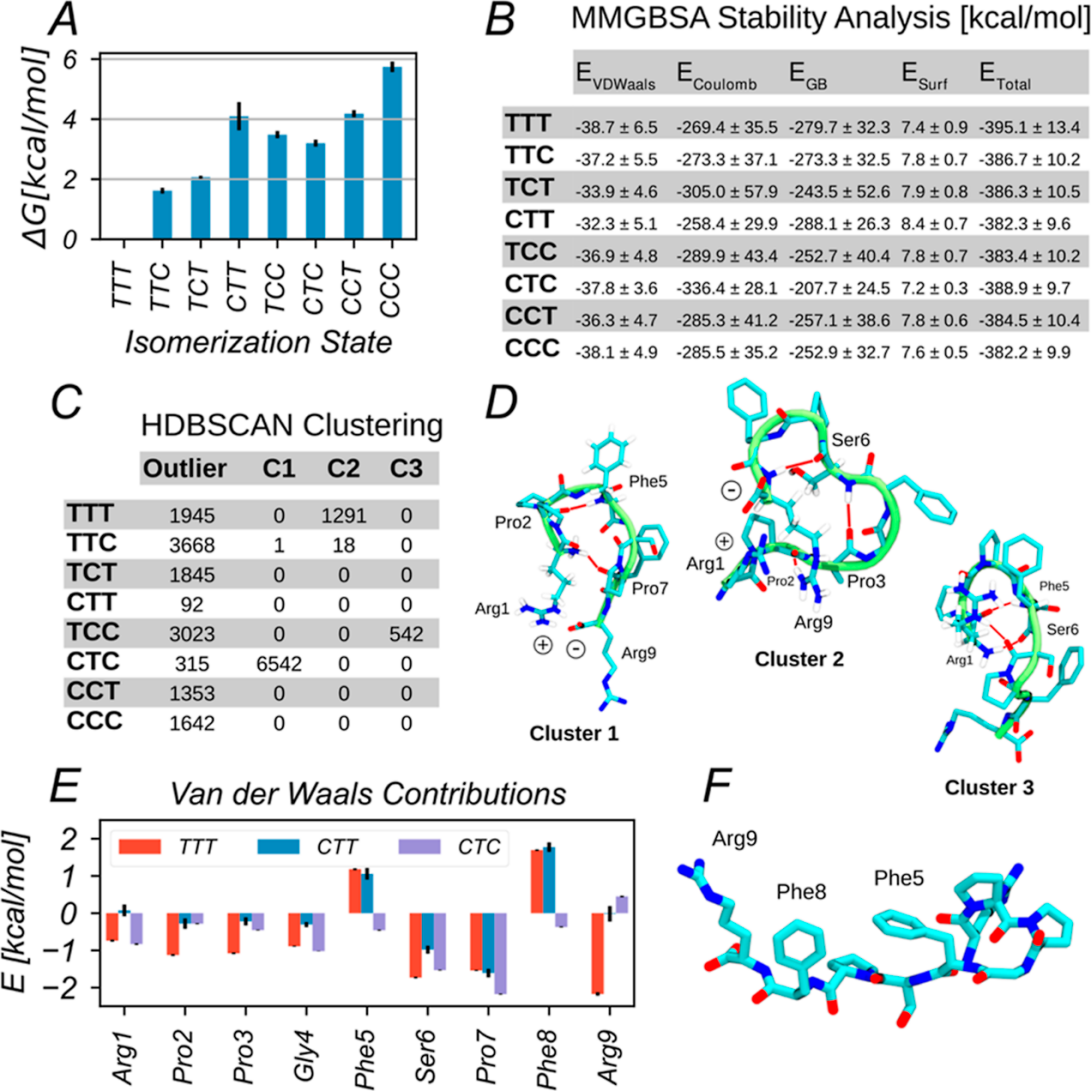
Free energies and representative conformations for the different isomerization states of BK. The isomerization states of BK are labeled TTT, TTC, TCT, CTT, TCC, CTC, CCT, and CCC, where the letters “T” and “C” refer to the trans and cis isomerization states, respectively, of single Pro residues, and the first, second, and third positions refer to Pro2, Pro3, and Pro7, respectively. (A) Free energies of the isomerization states relative to a value of zero for the all-trans state. Underlying values *G*(state) were obtained according to eq 7 (Section 2.2). Error bars refer to the standard error of the mean of subset free-energy values. (B) MMGBSA stability analysis of the isomerization states. *E*_VDWaals_, *E*_Coulomb_, *E*_GB_, and *E*_surf_ refer to the average molecular mechanics van-der-Waals and Coulomb energies as well as Generalized-Born electrostatic solvation-free energy and nonpolar surface area-based solvation-free energy, respectively. Differences in other energy terms like *E*_bond_, *E*_angle_, and *E*_dihedral_ are insignificant between the states and these energy terms are hence omitted from the table for better clarity. *E*_total_ is the sum of all energy terms. Errors refer to the standard error of the mean of subset quantities. (C) HDBSCAN clustering on the combined isomerization states sampled in replica 1. For each isomerization state, the number of members in the three most occupied clusters (C1, C2, and C3) is reported, along with the number of structures not present in any of those (“outliers”). (D) Representative configurations for clusters C1, C2, and C3 shown in stick representation. Important salt bridges and hydrogen bonds and the involved residues are highlighted. Backbone alignment is illustrated by green-tube representation. (E) Residue-wise decomposition of the MMGBSA van-der-Waals energy contribution *E*_VDWaals_ reported in panel (B) for selected isomerization states. Error bars refer to the standard error of the mean of subset energies. (F) Exemplary conformation of state CTT with unfavorable van-der-Waals energy, shown in stick representation. Important residues are highlighted.

The unbiased probability densities of sampling in replica 1 (eq 3; Section 2.2) suggest that zwitterionic BK in water mainly adopts the all-trans configuration with a probability of *P*(ω_2_ = ω_3_ = ω_7_ = 180°) = 89.99 ± 0.98%. The corresponding free energy is obtained from Boltzmann inversion (eq 7). It was here set to zero to express the free energies of the other isomerization states relative to the all-trans state. The resulting relative free energies for all states are listed in Figure 3A. These states are more unfavorable than the all-trans state by 1.62–5.75 kcal·mol^–1^, which is about 3–10 times the value of *k*_B_*T* at 300 K.

The all-trans state also populates configurations with the most favorable average interaction energies of *E* = −395.1 ± 13.4 kcal·mol^–1^, as shown by MMGBSA stability analysis (Figure 3B). Most notably, it is favorable solvent interactions that promote the all-trans state. Electrostatic BK-solvent interactions in the all-trans state are more favorable by 2–26% than in the other states, except CTT, which presents even more favorable solvent interactions (Figure 3B).

HDBSCAN clustering of important hydrogen-bond contacts identifies a cluster of similar configurations (cluster 2 in Figure 3C,D) in the highly diverse set of BK configurations, populated primarily by all-trans states and some TTC states. The latter is the state closest in free energy to the all-trans state (1.62 kcal·mol^–1^ more unfavorable; Figure 3A) and has a similarly favorable MMGBSA electrostatic solvation contribution (2% more unfavorable; Figure 3B). In the all-trans state, configurations fall into cluster 2 with a probability of ∼40%. Backbone rmsd values to cluster 2 are shown in Figure S2A.

Structures of cluster 2 are characterized by a salt bridge of the C-terminal carboxylate group to the positively charged guanidinium group of Arg1 as well as hydrogen-bond interactions between the backbone oxygen atom of Pro2 and the side chain guanidinium group of Arg9 (Figure 3D). Furthermore, frequent hydrogen-bond contacts between the backbone oxygen atom of Pro3 and the backbone amide hydrogen atom of Ser6, as well as between the backbone oxygen atom of Ser6 and the amide hydrogen atom of Arg9, are established in cluster 2 (Figure 3D). The latter hydrogen bond is present in 30.8% of configurations sampled in the all-trans state, while it is only present in 7.2% of simulation frames for state TCT, 4.3% for CTT, and 10.7% for CCT (using a looser donor–acceptor distance criterion of 3.5 Å; Section 2.4 and Table S1), and it is absent in the remaining isomerization states of BK. It is important in establishing the β-turn structure in the C-terminal residues Ser6-Arg9 of BK that was also found in in-solution NMR experiments of BK (Figures 3D and S2B).^15,17−19^ Our finding of a dominant all-trans population is supported by numerous NMR experiments,^18,19,33,57−59^ which allow the determination of a Pro isomerization state via the chemical shifts of Pro C_β_ and C_δ_ atoms.^60^

Furthermore, we analyzed the conformations sampled in replica 1 with respect to NOE upper bound violations using the experimental NOE intensities provided in ref (19) for zwitterionic BK in solution. This provides experimental validation for the conformations sampled in the all-trans state of BK in this study (Table 1). In particular, for a total of 30 NOE intensities, no violations were found for the conformations pertaining to the all-trans state. Also, no violations were found for the state CTT. Note, in this context, that none of the experimental NOEs involved protons of Pro2. However, NOEs involving both Pro3 and Pro7 were violated for states CCC, TCC, and TCT, NOEs involving Pro3 only were violated for state CCT, and NOEs involving Pro7 only were violated for states CTC and TTC (Table 1). This may be an additional indication that the experimental ensemble of structures presents Pro3 and Pro7 in the trans states.

**Table 1:** NOE Upper Bound Violations Δ_*ij*_ (eq 14) for Different Isomerization States of BK^a^

state	atoms		NOE intensity	Δ_*ij*_ [Å]
CCC	Phe5, H	Pro3, HA	vw	0.6
	Arg9, H	Pro7, HA	vw	0.1
CCT	Phe5, H	Pro3, HA	vw	0.7
CTC	Phe8, H	Pro7, HD2^b^	w	1.4
TCC	Phe5, H	Pro3, HA	vw	0.6
	Arg9, H	Pro7, HA	vw	0.2
TCT	Phe5, H	Pro3, HA	vw	0.8
	Arg9, H	Pro7, HA	vw	0.2
TTC	Arg9, H	Pro7, HA	vw	0.3

a The isomerization states of BK are labeled TTT, TTC, TCT, CTT, TCC, CTC, CCT, and CCC, where the letters “T” and “C” refer to the trans and cis isomerization states, respectively, of single Pro residues, and the first, second, and third positions refer to Pro2, Pro3, and Pro7, respectively. No violations are observed for states CTT and TTT. NOE intensities are given as strong (s), medium (m), weak (w), or very weak (vw), and corresponding experimental upper bounds^19^ were 3, 3.5, 4, or 4.5 Å, respectively.

b Stereospecific assignment to HD2 was made based on violations observed for HD3 in all sampled states.

Besides the all-trans state, the TTC and TCT states are populated with unbiased probabilities of 6.18 ± 0.81% and 2.82 ± 0.21%, respectively. These states are closest in free energy to the all-trans state (Figure 3A). MMGBSA stability analysis does not reflect a clear energetic preference of state TTC compared to other nonall-trans states, except for the electrostatic solvation-free energy contribution that is of similarly low magnitude as that for the all-trans state (Figure 3B). Favorable solvation or entropic contributions omitted in the MMGBSA analysis may hence play a role in stabilizing the TTC state. Note that only a tiny fraction of TTC configurations (0.5%) could be assigned to certain conformational clusters (Figure 3C).

The CTT state is significantly less probable, with a probability of 0.19 ± 0.09%. While it displays the most favorable MMGBSA electrostatic solvation-free energy, it has particularly unfavorable intrapeptide nonpolar interactions (Figure 3B). Notably, the cis isomer of Pro2, as it occurs in state CTT, appears to be significantly more unfavorable than the cis isomer of Pro3 or Pro7 (e.g., as occurring in states TCT and TTC). This may be traced back to unfavorable van-der-Waals interactions involving Phe5 and Phe8 (Figure 3F). As indicated by the residue-wise decomposition of MMGBSA energy contributions, these residues have particularly high van-der-Waals energy contributions in states TTT and CTT, whereas their van-der-Waals energy contributions are favorable in state CTC (Figure 3E). This is also reflected in the average van-der-Waals interaction energy between the two residues, which is among all of the BK isomer states the most favorable in state CTC (−2.0 kcal·mol^–1^; Table S2). In states TTT and CTT, however, the favorable Phe5-Phe8 van-der-Waals interaction is missing (Table S2).

The unfavorable intrapeptide energies observed in the state CTT can be largely ameliorated by the isomerization of a second Pro residue, Pro7. In fact, the CTC state of BK has the third lowest relative free energy with respect to the all-trans state and the second lowest MMGBSA total energy in addition to the all-trans state. The stability of state CTC is particularly reflected in a low average Coulomb energy of −336.4 ± 28.1 kcal·mol^–1^ at expense of somewhat more unfavorable solvent interactions (Figure 3B).

Conformational clustering shows that state CTC is highly structured, with almost all (∼95%) sampled configurations falling into cluster 1. This cluster appears structurally very homogeneous (Figure S1B). Backbone rmsd values to cluster 1 are shown in Figure S1A. In cluster 1, a very stable salt bridge is formed between the C-terminal carboxylate group and the positively charged guanidinium group of Arg1 (Figure 3D). This is in keeping with the low Coulomb energy of the state CTC (Figure 3B). However, as suggested by the structural homogeneity of cluster 1 (Figure S1B), unfavorable conformational entropy contributions may act to destabilize state CTC. Hydrogen bonding between the backbone oxygen atom of Pro2 and the backbone amide hydrogen atom of Phe5 as well as between the backbone oxygen atom of Pro7 and the positively charged N-terminal amino group further stabilizes the conformation.

A smaller cluster 3 populated by conformations of state TCC was found. Cluster 3 appears to be structurally very homogeneous (Figure S3B). It features frequent hydrogen bonding between the backbone oxygen atom of Arg1 and the backbone amide hydrogen atom of Phe5, as well as between the backbone oxygen atom of Ser6 and the positively charged N-terminal amino group and the backbone oxygen atom of Phe5 and the guanidinium group of Arg1. Backbone rmsd values to cluster 3 are shown in Figure S3A.

### 3.3 Cis–Trans Isomerization Equilibria of All Pro Residues in BK

**Figure 4:**
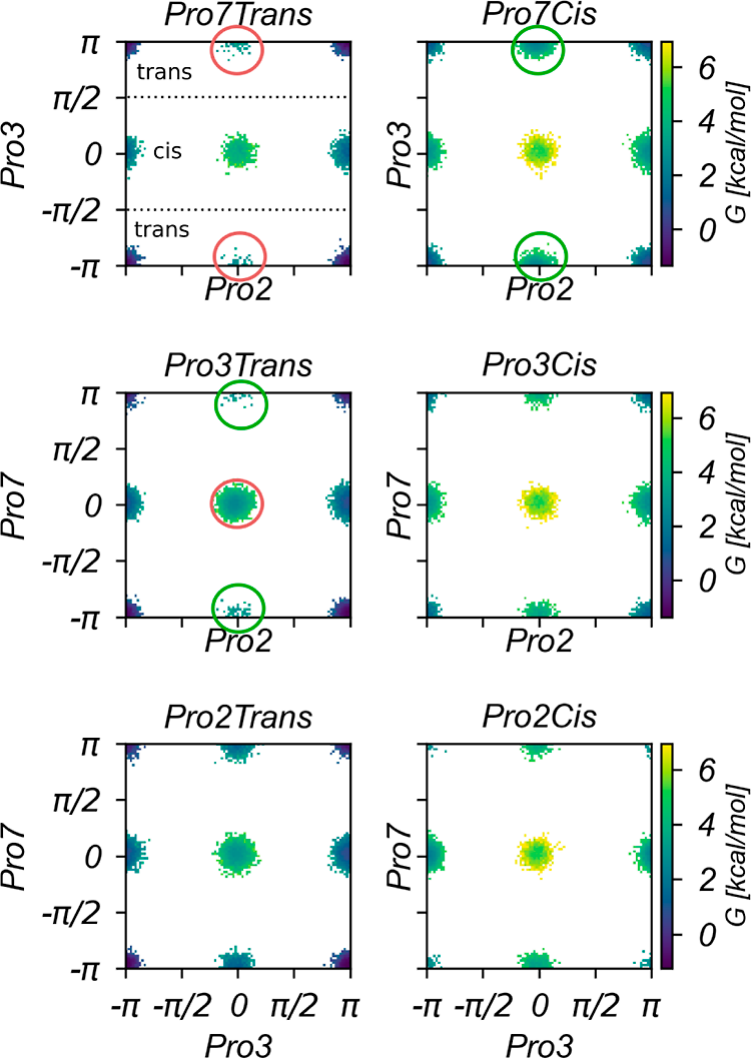
Two-dimensional free-energy landscapes as function of the ω dihedral angles of two Pro residues *i* and *j*, indicated on the *x*- and *y*-axes, of BK. Free energies were obtained from Boltzmann inversion of the corresponding unbiased probabilities (eq 8). They depend on the isomerization state of the third Pro residue *k*, which is indicated on top of each graph. Highlighted regions show that Pro2 is significantly more likely to be found in the cis isomer, if Pro7 also adopts the cis isomer.

Based on a two-dimensional projection of the three-dimensional unbiased probability densities (eq 8; Section 2.2), two-dimensional free-energy surfaces were calculated via Boltzmann inversion (Figure 4). This allows for visual detection of the coupling of isomerization events. For instance, Pro2 appears to be significantly more likely to be found in the cis isomer if Pro7 is also in the cis isomer. This is consistent with the above-noted observation that BK conformations achieved by isomerization of Pro7 to the cis state (as in state CTC) appear to be much more favorable than those compatible with only Pro2 being in the cis state (as in state CTT; Section 3.2).

**Figure 5:**
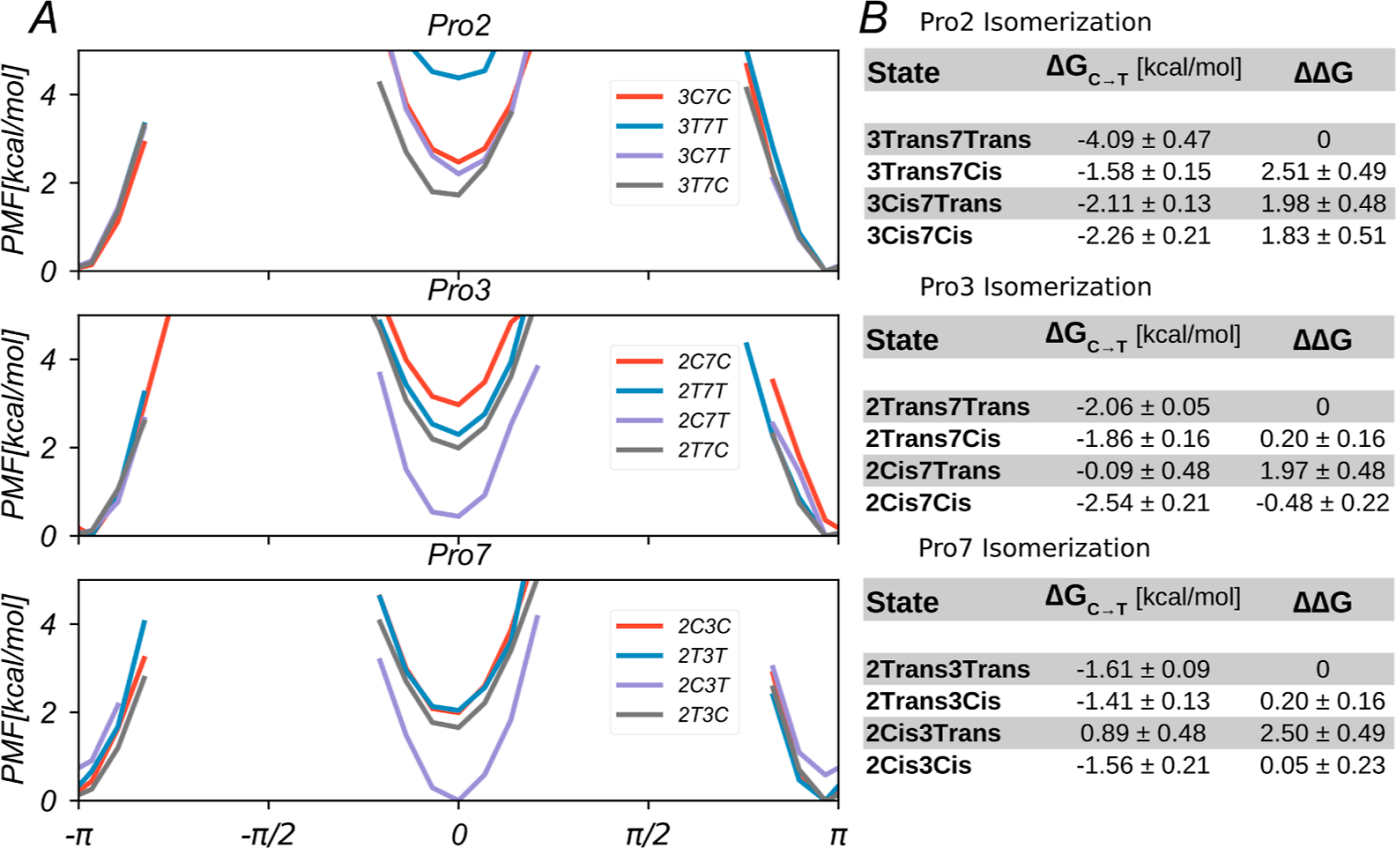
Conditional free-energy profiles and isomerization free energies for BK. (A) Free-energy profiles as function of the ω dihedral angle of a Pro residue *i*, indicated on top of each graph. Free energies were obtained from Boltzmann inversion of the corresponding unbiased probabilities (eq 9). They depend on the isomerization state of the other two Pro residues *j* and *k*, which are indicated as labels to each PMF, where numbers 2, 3, and 7 denote the Pro residue and letters “T” and “C” denote the trans or cis state for the Pro residue. (B) Conditional isomerization free energies Δ*G*_cis→trans_ (eq 12) for Pro residues 2, 3, and 7 (from top to bottom). The isomerization state of the other two Pro residues is indicated in bold font. The quantity ΔΔ*G* describes how the conditional isomerization free energy changes when any of the other two Pro residues switches from the trans to the cis state. Errors refer to the standard error of the mean of subset quantities.

By calculating PMFs along the ω dihedral angles of each Pro residue of BK in dependence of the other Pro isomerization states, we can systematically and in detail quantify the mutual influence of Pro isomerization states on the isomerization equilibria in BK via conditional isomerization free energies (eq 12; Section 2.3). These show that the isomerization processes in BK are not independent (Figure 5A).

For instance, for Pro2, 4.09 kcal·mol^–1^ have to be invested when switching from the trans to the cis state if both Pro3 and Pro7 are in the trans state, but only 1.58 kcal·mol^–1^ have to be invested when Pro3 is in the trans state and Pro7 is in the cis state. Interestingly, when Pro3 is in the cis state, the isomerization state of Pro7 has almost no influence on the isomerization free energy of Pro2 (Figure 5B, top).

Pro3 shows the least discrimination between the cis and trans states when Pro2 is in the cis state and Pro7 is in the trans state. In this case, the difference between the Pro3 cis and trans states is only −0.09 kcal·mol^–1^. Otherwise, switching from the cis to the trans state involves a free energy gain of −2.54, −2.06, and −1.86 kcal·mol^–1^ for states where both Pro2 and Pro7 are in the cis and trans states, or Pro2 is in the trans and Pro7 in the cis state, respectively (Figure 5B, middle).

Of note, Pro7 is more likely to be found in the cis isomer when Pro2 is in the cis isomer and Pro3 is in the trans isomer, with a free energy of isomerization of 0.89 kcal·mol^–1^ for switching to the trans isomer. This appears to be the only conditional isomerization equilibrium of zwitterionic BK in water that favors the cis state of a Pro residue. In contrast, other cis/trans combinations of Pro2 and Pro3 entail a more favorable trans state of Pro7, with free energy gains of −1.41 to −1.61 kcal·mol^–1^ (Figure 5B, bottom).

All Pro residues *i* considered, the differences in conditional isomerization free energies with respect to Pro residues *j* and *k* being in the trans state span a range of −0.48 to 2.51 kcal·mol^–1^ (Figure 5B). Isomerization equilibria can hence be drastically influenced by the isomerization states of the other Pro residues. To the best of our knowledge, this is the first time that a strong coupling of multiple Pro isomerization states has been systematically illustrated and quantified via free-energy calculations. The fact that such coupling is observed in BK, a short and highly flexible peptide with a partial random coil-like character, is remarkable. Especially since two of its Pro residues (Pro2 and Pro3) are located in the unstructured N-terminal region (Arg1-Phe5), one may assume that coupling of Pro isomerization states may play a similar, if not more decisive, role in systems where conformational restraints are present, as, for example, in folded proteins. For instance, stable secondary structure elements can be adversely affected by the isomerization of a given Pro residue, and their destabilization may facilitate the isomerization of another Pro residue. On the other hand, isomerization of a given Pro residue to the native state, as in a folding process, may stabilize certain secondary structure elements and concomitantly also favor the isomerization state of another Pro residue, which is more compatible with the present structural restraints.

## 4 Conclusions

Pro cis/trans isomerization can be an important determinant in the protein folding process^4^ and can underlie intricate biological phenomena such as molecular timing.^3^ This is because in nature, Pro cis/trans isomerization has a very high energy barrier of around 20 kcal·mol^–1^ and occurs on tremendous time scales of seconds to minutes. These time scales, although intimately connected to the biological significance of Pro isomerization, are not accessible in atomistic explicit-solvent MD simulations, which is why the ωBP-REMD method was used in the present work to investigate Pro cis/trans isomerization.

ωBP-REMD, which was developed in previous work,^7^ is an enhanced-sampling method based on Hamiltonian REMD that allows frequent sampling of cis/trans transitions of given Pro residues in a manner that achieves approximately equal occupations of the cis and trans isomers. Isomerization free energies are calculated via Boltzmann inversion of unbiased probabilities.

Besides its computational efficiency arising from equally focusing the sampling in the lowermost replica on the cis and trans isomerization states, an advantage of ωBP-REMD is its power in the investigation of systems where multiple Pro residues occur. Rather than having to sample the volume of the multidimensional conformational space spanned by multiple combined reaction coordinates as in, for example, metadynamics, ωBP-REMD biases independently the multiple reaction coordinates. Hence, when several reaction coordinates are considered simultaneously, ωBP-REMD requires considerably less sampling effort compared to standard metadynamics or umbrella sampling approaches.

In the present work, the studied system featuring multiple Pro residues was zwitterionic aqueous BK. The peptide is partially unstructured and has three Pro residues, whose isomerization equilibria were calculated. In particular, the raw outcome of the performed ωBP-REMD simulation, i.e., biased probabilities of isomerization states of BK, was unbiased and analyzed in terms of three-, two-, and one-dimensional free-energy surfaces.

The conditional isomerization free energy of a Pro residue *i*, given by the free-energy difference of the cis and trans states of this residue in dependence of the isomerization state of the two other Pro residues *j* and *k* in BK was calculated for all Pro residues of BK. Thus, comprehensive quantitative insight into the coupling of isomerization states can be obtained. Furthermore, the sampled conformations were analyzed in detail and compared to experimental NMR data.

ωBP-REMD did not only give excellent cis/trans transition statistics for all three BK Pro residues but also allowed access to all eight possible BK isomerization states with converged probabilities during a simulation length of 885 ns.

Important findings are as follows:

1. The all-trans state is found to be the preferred state. The preference for the all-trans state of aqueous zwitterionic BK has been validated by several experimental NMR studies.^18,19,33,57−59^ In the present study, an analysis of NOE upper bound violations further supported the conformational sampling of the all-trans state.
2. Distinct conformational clusters are sampled by the eight different isomerization states of BK. Of note, in about one-third of the simulation frames, the all-trans state presents the characteristic Ser6-Arg9 hydrogen bond forming the experimentally confirmed^15,17−19^ C-terminal β-turn motif. The cluster predominantly sampled by state CTC appears highly structured. While state CTC is about 3.3 kcal·mol^–1^ less favorable than the all-trans state, it involves profound stabilization by favorable intrapeptide electrostatic interactions.
3. Isomerization of the three Pro residues in BK can occur in a coupled fashion, i.e., the isomerization state of a given Pro residue can influence the cis/trans equilibrium of other Pro residues. We quantify the influence of the isomerization state of two Pro residues *j* and *k* on the isomerization equilibrium of Pro residue *i* with conditional isomerization free energies, which are the difference in the free energies of the cis and trans isomerization state of Pro *i* for the different combinations of isomerization states of Pro residues *j* and *k*. These free energies are conveniently obtained from the projection of the three-dimensional (unbiased) probability density obtained from replica 1 of a ωBP-REMD simulation on the reaction coordinate of interest, i.e., the ω dihedral angle of Pro *i*. A notable example for BK is that Pro7 is more likely to be found in the cis isomer than in the trans isomer when Pro2 is in the cis isomer (and Pro3 is in the trans isomer). This appears to be the only conditional isomerization equilibrium of zwitterionic BK in water that favors the cis state of a Pro residue.
4. The influence of Pro isomerization states on the cis/trans equilibrium of other Pro residues may be large, i.e., conditional isomerization free energies may differ by up to 2.5 kcal·mol^–1^.

While it was previously noted that the isomerization states of the individual Pro residues in BK appear not to be independent,^12^ to our knowledge, the present work is the first time that a strong coupling of Pro isomerization states has been systematically illustrated and quantified via extensive free-energy calculations. It is noteworthy that such coupling is observed in such a small and unstructured system as BK. This suggests that coupling of Pro isomerization states may play a similar, if not more significant, role in systems exhibiting more conformational restraints like folded proteins.

## Data Availability

Coordinates of the configurations in clusters 1–3 are available at 10.5281/zenodo.10566361. Basic scripts to perform and analyze ωBP-REMD simulations with AMBER are provided in an online repository.^61^

## Supporting Information Available

Characterization of the eight different isomerization states of BK with respect to sampling in clusters 1–3; hydrogen bonds in clusters 1–3; and average van-der-Waals interaction energies between residues Phe5 and Phe8 of BK isomer states (PDF)
