# Supplementary material for: Comprehensive Analysis of Coupled Proline Cis–Trans States in Bradykinin Using ωBP-REMD Simulations
Source: J Chem Theory Comput. 2024 Mar 11;20(6):2643–54. doi: 10.1021/acs.jctc.3c01356 (PMC10976632; doi:10.1021/acs.jctc.3c01356)
Supplement: Supplementary file 1 — ct3c01356_si_001.pdf [file ct3c01356_si_001.pdf]

Supplementary Material

Comprehensive analysis of coupled proline cis-trans states in bradykinin using ωBP-REMD simulations

Maximilian Kienlein[1], Martin Zacharias[1] and Maria M. Reif[1,*]

[1] *Center for Functional Protein Assemblies (CPA), Physics Department, Chair of Theoretical Biophysics (T38), Technical University of Munich, Ernst-Otto-Fischer-Str. 8, 85748 Garching, Germany*

---

*Corresponding author:
Dr. Maria Reif,
Center for Functional Protein Assemblies (CPA),
Physics Department,
Chair of Theoretical Biophysics (T38),
Technical University of Munich,
Ernst-Otto-Fischer-Str. 8,
85748 Garching, Germany
Phone: +49 89 289 12731
e–mail: maria.reif@tum.de

February 12, 2024

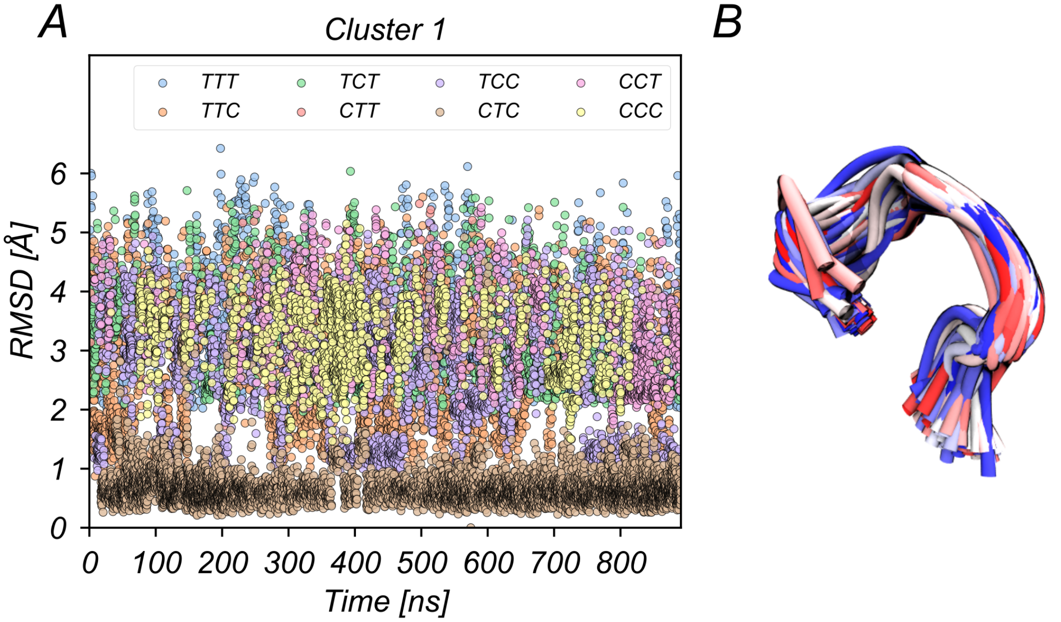

Figure S1: Characterization of the eight different isomerization states of bradykinin (BK) with respect to sampling in cluster 1 and illustration of structures sampled in cluster 1. The underlying clustering was done in a combined fashion on the entire trajectory of replica 1 (Section 2.4 of the main article). (A) Root mean square deviation of the BK C_α atoms with respect to the configuration closest to the centroid of cluster 1, evaluated after a rototranslational fit to these atoms. The data is depicted separately for the eight BK isomers TTT, TTC, TCT, CTT, TCC, CTC, CCT and CCC. Here, the first, second and third letters refer to the trans ("T") or cis ("C") states of Pro2, Pro3, and Pro7, respectively. The trajectories pertaining to these isomerization states were filtered out from replica 1 of the ωBP-REMD simulation and contained 3236, 3687, 1845, 92, 3565, 6857, 1353 and 1642 frames, respectively. Each dot represents a simulation frame. (B) Backbone superposition of the members of cluster 1. For clarity of visual illustration, only every 20th frame along the trajectory containing the members of this cluster is shown. Colorcoding from blue to red indicates the timestep of the trajectory (dark blue, white and dark red indicating the beginning, middle and end of the trajectory, respectively).

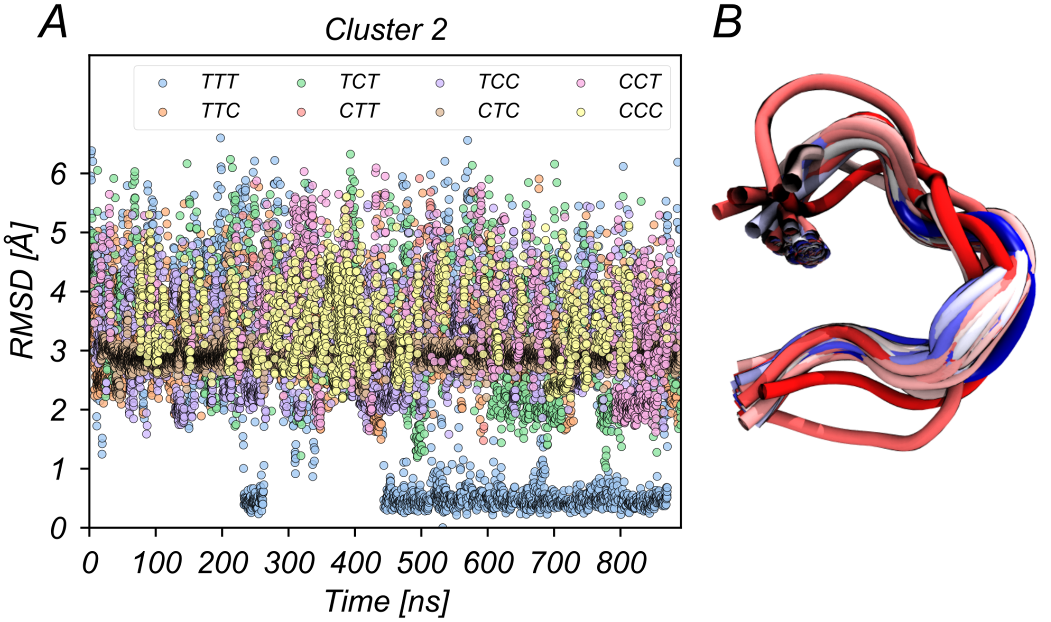

Figure S2: Characterization of the eight different isomerization states of bradykinin (BK) with respect to sampling in cluster 2 and illustration of structures sampled in cluster 2. The underlying clustering was done in a combined fashion on the entire trajectory of replica 1 (Section 2.4 of the main article). (A) Root mean square deviation of the BK C_α atoms with respect to the configuration closest to the centroid of cluster 2, evaluated after a rototranslational fit to these atoms. The data is depicted separately for the eight BK isomers TTT, TTC, TCT, CTT, TCC, CTC, CCT and CCC. Here, the first, second and third letters refer to the trans ("T") or cis ("C") states of Pro2, Pro3, and Pro7, respectively. The trajectories pertaining to these isomerization states were filtered out from replica 1 of the ωBP-REMD simulation and contained 3236, 3687, 1845, 92, 3565, 6857, 1353 and 1642 frames, respectively. Each dot represents a simulation frame. (B) Backbone superposition of the members of cluster 2. For clarity of visual illustration, only every 10th frame along the trajectory containing the members of this cluster is shown. Colorcoding from blue to red indicates the timestep of the trajectory (dark blue, white and dark red indicating the beginning, middle and end of the trajectory, respectively).

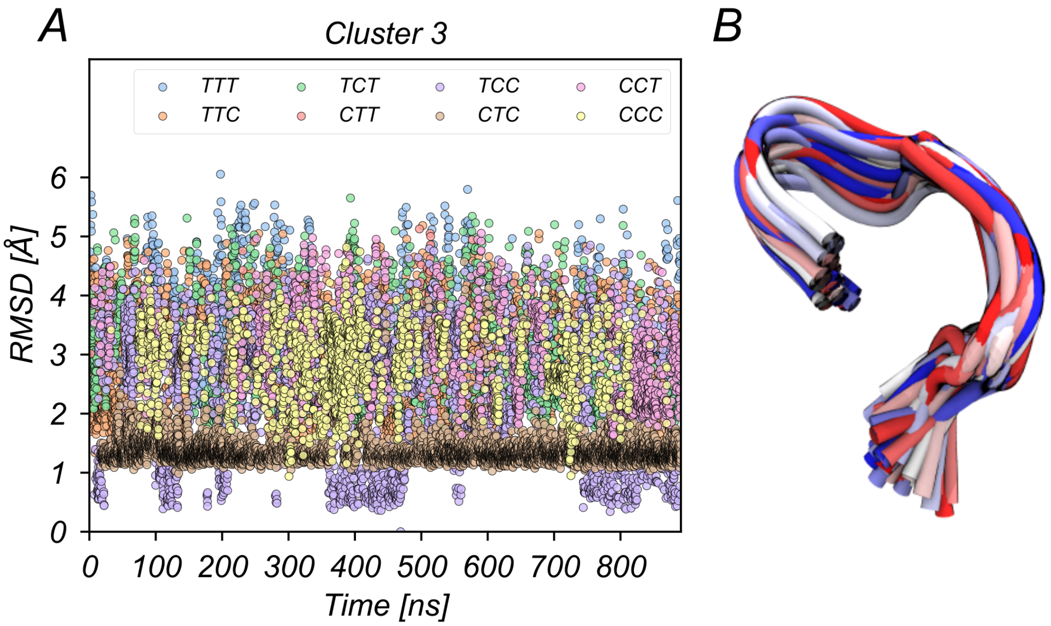

Figure S3: Characterization of the eight different isomerization states of bradykinin (BK) with respect to sampling in cluster 3 and illustration of structures sampled in cluster 3. The underlying clustering was done in a combined fashion on the entire trajectory of replica 1 (Section 2.4 of the main article). (A) Root mean square deviation of the BK C_α atoms with respect to the configuration closest to the centroid of cluster 3, evaluated after a rototranslational fit to these atoms. The data is depicted separately for the eight BK isomers TTT, TTC, TCT, CTT, TCC, CTC, CCT and CCC. Here, the first, second and third letters refer to the trans ("T") or cis ("C") states of Pro2, Pro3, and Pro7, respectively. The trajectories pertaining to these isomerization states were filtered out from replica 1 of the ωBP-REMD simulation and contained 3236, 3687, 1845, 92, 3565, 6857, 1353 and 1642 frames, respectively. Each dot represents a simulation frame. (B) Backbone superposition of the members of cluster 3. For clarity of visual illustration, only every 5th frame along the trajectory containing the members of this cluster is shown. Colorcoding from blue to red indicates the timestep of the trajectory (dark blue, white and dark red indicating the beginning, middle and end of the trajectory, respectively).

acceptor atom	donor hydrogen atom	donor atom	fraction [%]
	state CCT		
SER6@OG	PHE8@H	PHE8@N	44.7
	state CTC		
PRO2@O	PHE5@H	PHE5@N	82.6
ARG9@O	ARG1@HE	ARG1@NE	37.4
ARG9@OXT	ARG1@HE	ARG1@NE	36.1
ARG9@OXT	ARG1@HH21	ARG1@NH2	31.4
ARG9@O	ARG1@HH21	ARG1@NH2	27.7
	state CTT		
PRO2@O	PHE5@H	PHE5@N	48.9
SER6@OG	PHE8@H	PHE8@N	35.9
	state TCT		
SER6@OG	PHE8@H	PHE8@N	34.9
ARG9@OXT	ARG1@HE	ARG1@NE	28.1
	state TTT		
SER6@OG	PHE8@H	PHE8@N	56.8
PRO3@O	SER6@H	SER6@N	40.1
SER6@O	ARG9@H	ARG9@N	30.8
PRO2@O	ARG9@HH11	ARG9@NH1	30.3
ARG9@OXT	ARG1@HE	ARG1@NE	28.8
ARG9@OXT	ARG1@HH21	ARG1@NH2	28.2
	cluster 1		
PRO2@O	PHE5@H	PHE5@N	84.7
ARG9@O	ARG1@HE	ARG1@NE	38.3
ARG9@OXT	ARG1@HE	ARG1@NE	37.3
ARG9@OXT	ARG1@HH21	ARG1@NH2	31.9
ARG9@O	ARG1@HH21	ARG1@NH2	28.8
	cluster 2		
PRO3@O	SER6@H	SER6@N	94.6
SER6@OG	PHE8@H	PHE8@N	78.6
PRO2@O	ARG9@HH11	ARG9@NH1	74.1
ARG9@OXT	ARG1@HE	ARG1@NE	67.9
ARG9@OXT	ARG1@HH21	ARG1@NH2	66.2
SER6@O	ARG9@H	ARG9@N	55.0
	cluster 3		
ARG1@O	PHE5@H	PHE5@N	86.4
SER6@O	ARG1@HE	ARG1@NE	42.4
PHE5@O	ARG1@H2	ARG1@N	36.2
PHE5@O	ARG1@H1	ARG1@N	34.1

Table S1: Hydrogen bonds present in BK isomers CCT, CTC, CTT, TCT and TTT, or in clusters 1, 2 and 3 (Section 2.4 of the main article). In the BK state specification, the first, second and third letters refer to the trans ("T") or cis ("C") states of Pro2, Pro3, and Pro7, respectively. Here, hydrogen bonds were defined via a donor-acceptor distance of less than 3.5 Å and a donor-hydrogen-acceptor angle of greater than 135°. Only hydrogen bonds with an occurrence in at least 25% of the respective simulation frames are listed and the corresponding fraction of occurrence is reported. For BK states CCC, TCC and TTC, no hydrogen bonds were found with a minimum occurrence of 25%. Atom name specifications refer to the nomenclature used in the AMBER topology.[2]

state	VDW energy [kcal·mol^{-1}]
TTT	-0.2 ± 0.01
CTT	-0.2 ± 0.02
CTC	-2.0 ± 0.02
CCC	-1.9 ± 0.07
CCT	-0.2 ± 0.02
TCC	-1.7 ± 0.06
TCT	-0.2 ± 0.02
TTC	-1.8 ± 0.02

Table S2: Average van-der-Waals (VDW) interaction energies between residues Phe5 and Phe8 of BK in the eight possible BK isomer states. In the BK state specification, the first, second and third letters refer to the trans ("T") or cis ("C") states of Pro2, Pro3, and Pro7, respectively. Statistical errors are calculated based on block averaging.[1]

References

[1] M.P. Allen and D.J. Tildesley. *Computer simulation of liquids.* Oxford University Press, New York, USA, 1987.

[2] D.A. Case, K. Belfon, I.Y. Ben-Shalom, S.R. Brozell, D.S. Cerutti, T.E. Cheatham III, V.W.D. Cruzeiro, T.A. Darden, R.E. Duke, G. Giambasu et al., AMBER 2020, University of California, San Francisco, `https://ambermd.org/`, accessed 2020
